# Robotic Cane Controlled to Adapt Automatically to Its User Gait Characteristics

**DOI:** 10.3389/frobt.2020.00105

**Published:** 2020-08-12

**Authors:** Andrés Trujillo-León, Ragou Ady, David Reversat, Wael Bachta

**Affiliations:** CNRS, UMR 7222, INSERM, U1150, Institut des Systèmes Intelligents et de Robotique, Sorbonne Université, Paris, France

**Keywords:** assistive devices, robotic cane, gait cycle, synchronization, mobility

## Abstract

Research on robotic assistance devices tries to minimize the risk of falls due to misuse of non-actuated canes. This paper contributes to this research effort by presenting a novel control strategy of a robotic cane that adapts automatically to its user gait characteristics. We verified the proposed control law on a robotic cane sharing the main shape features of a non-actuated cane. It consists of a motorized telescopic shaft mounted on the top of two actuated wheels driven by the same motor. Cane control relies on two Inertial Measurement Units (IMU). One is attached to the cane and the other to the thigh of its user impaired leg. During the swing phase of this leg, the motor of the wheels is controlled to enable the tracking of the impaired leg thigh angle by the cane orientation. The wheels are immobilized during the stance phase to provide motionless mechanical support to the user. The shaft length is continuously adjusted to keep a constant height of the cane handle. The primary goal of this work is to show the feasibility of the cane motion synchronization with its user gait. The control strategy looks promising after several experiments. After further investigations and experiments with end-users, the proposed control law could pave the road toward its use in robotic canes used either as permanent assistance or during rehabilitation.

## 1. Introduction

Falls are a major health, societal and economic problem, resulting in 424,000 annual fatalities around the world (World Health Organization, 2012). When they are not fatal, they cause high traumas and morbidity. Indeed, falling may result in damages ranging from minor bruises to more serious brain injuries and hip fractures (Sterling et al., 2001). In Europe, falls related costs are estimated at 25 billion euros each year (European Public Health Association, 2015).

The most common response to falls is the use of assistive devices. Even though they are perceived positively by the patients (Tyson and Rogerson, 2009), the improper use of these devices is among the extrinsic causes leading to falls (Liu et al., 2011).

In the last two decades, many lines of research have been dedicated to improving the assistance provided by assistive devices. Mainly, instrumented and robotic devices have been developed. For instance, instrumented canes, which consist in canes equipped with strain gauges, inertial measurement units (IMUs), embedded computers and other equipment have been designed to enable an unobtrusive monitoring of cane use (Au et al., 2008; Mercado et al., 2014; Trujillo-León et al., 2015; Wade et al., 2018). Normally, the cane orientation and the forces applied to it are measured and collected to enable their later analysis by the medical staff.

Robotic canes, on the other hand, aim to provide additional assistive functionalities and they generally share the same mechanical architecture. They consist in a basic cane mounted on the top of a statically stable wheeled mobile robot. The additional functions provided by robotic canes include navigation assistance, user intention detection or fall prevention. In Spenko et al. (2006), navigation assistance functions are provided. The mobile platform consists of two driving and one castor wheels. It encloses a camera and a sonar array used for localization purposes. The cane mounted on the top of this platform is equipped with a force and torque sensor that measures the load applied by the user. In the first navigation mode, the robotic cane, using its localization system, moves autonomously to guide the user toward a desired position. In the second mode, a shared control is implemented, i.e., the user moves the robotic cane but the latter can correct his deviations from a pre-planned path.

Robotic canes come with extra weight resulting mainly from the integrated batteries and structure. Some robotic canes detect the walking direction and move accordingly, thus alleviating the user muscular effort. In Wakita et al. (2013), a cane enclosing a force and torque sensor is mounted on the top of an omnidirectional mobile platform. The device is controlled through an on-line estimation of the Intentional Direction (ITD). The ITD is estimated using the direction of the horizontal force applied to the cane by its user. In order to secure the user gait, the cane controller is tuned to make it move easily along the detected direction and to be difficult to maneuver in the other directions.

To prevent falls, robotic canes adapt their motion to their user balance. In Suzuki et al. (2009), a cane is mounted on a mobile base that consists of two wheels with servo brakes and two castor wheels. The platform is equipped with laser range finders. Indeed, the brake control is adapted to the distance between the user feet position and the robotic cane. In Di et al. (2016), a robotic cane is controlled in a way that avoids its users tipping over when holding it. A recent work is presented in Phi and Fujimoto (2019); an innovative robotic cane incorporating an omnidirectional motorized wheel has been proposed.

In previous works (Ady et al., 2013, 2014), we proposed a prototype of robotic cane that avoided the common bulky and cumbersome structure of robotics canes and walkers, and that shared the compactness and longitudinal shape that characterize the conventional ones. In this paper, we focus on the control strategy using a revisited version of the robotic cane. The control law is aimed at providing safe and proper support in the very instants it is required. For that purpose, the cane motion is automatically synchronized with its user gait, without requiring any specific intervention.

### 1.1. Synchronization of a Cane With the Gait Cycle

**Figure 1:**
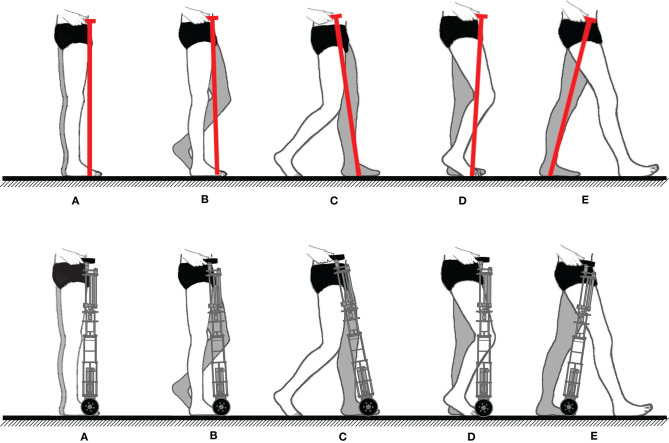
(Top) Sequential representation of contralateral cane assisted walk (weakest leg grayed). **(A)** Person standing. **(B)** Weakest leg and cane forward swing. **(C)** Weakest leg and cane stance beginning. **(D)** Weight support performed by the weakest leg and the cane. **(E)** Beginning of the sound leg stance. (Bottom) Robotic cane synchronization desired during the gait (weakest leg grayed). In **(C)**, coinciding with the impaired leg heel strike, the wheels stop. In **(E)**, the cane starts moving again with the same leg push off.

People use their arm to synchronize their basic cane motion with their gait cycle during straight forward walking. This synchronization can be analyzed in the sagittal plane. Only one stride is required since walking straight forward is cyclic. As depicted in Figure 1 top, starting from a standing position (a), the weak leg (grayed in the figure) leaves the ground and starts its swing phase (b), the cane is lifted and moved forward in synergy with the leg motion. The cane tip is put on the ground a step further and synchronously with the impaired leg heel strike (c). The maximum tangential forces applied to the cane occur during the heel-strike (c) and the push off (e) of the impaired leg. The maximum normal force applied to the cane occurs during the phase (d) (Chen et al., 2001).

The robotic cane, which design is detailed in the next section, is aimed at making this synchronization automatic, i.e., the user no longer needs to lift the cane at each step. Instead, it should adapt automatically its motion to the gait cycle. To provide the same assistance of a conventional cane, its wheels should move forward during the impaired leg swing phase and stop when the latter touches the ground. At the same time, the shaft length should vary continuously to keep a constant height of the cane handle in order to avoid pushing or pulling the user hand. The intended synchronization scheme of the robotic cane is depicted in Figure 1 bottom.

### 1.2. Synchronization of the Cane Wheels With the Impaired Leg Motion

If the step size and its duration are learned offline, the motion of the wheels can be achieved in open loop, i.e., a predefined trajectory could be programmed. In this case, the cane motion cannot adapt to changes of the gait parameters. However, the objective here is to enable the cane adapting to its user's gait characteristics.

An alternative choice, consisting in a closed loop control of the displacements of the wheels based on motion synergies, is preferred. Motion synergies have already been used in rehabilitation robotics to generate reference trajectories for exoskeletons. For instance, the authors of Vallery et al. (2009) took advantage from the existing synergy between lower limb joints to provide reference trajectories to an exoskeleton assisting an impaired limb based on the motion of the sound leg. Synchronizing robotic motion with respect to a cane assisted gait cycle has been studied in Hassan et al. (2012); Hassan et al. (2018). In their study, the authors used the existing cane-lower limbs synergy to control a single leg version of the HAL exoskeleton. Firstly, they assessed the existence of a coordination between the lower limb joints trajectories and the cane angle (the cane rotation in the sagittal plane). Then, they implemented a limb motion estimation method, i.e., the cane angle and that of the sound leg joints were used to generate the reference motion of the exoskeleton assisting the impaired leg.

In our work, the aim is to control the robotic cane motion based on the impaired leg motion. Moreover, unlike the setup used in Hassan et al. (2012), we would like to reduce the required components by equipping the user with only one IMU.

The paper is organized as follows. In section 2, the current prototype is presented. The synchronization strategy is presented and supported by experimental results in section 3. In section 4, the control law of the cane is derived. In section 5, the experimental results, obtained using the prototype and its associated control law, are discussed. A conclusion ends the paper by giving some future research directions.

## 2. Robotic Cane Presentation

In this section, the design objectives of the robotic cane are given. Its mechanical architecture, as well as its embedded electronics, are then presented.

### 2.1. Design Objectives

From a mechanical point of view, the goal is providing a lightweight compact cane able to follow the pace of people with balance troubles. The prototype, presented hereafter, is based on requirements expressed in terms of the cane forward velocity and support forces.

In Chen et al. (2001), the mean pace of 20 post-stroke hemiplegic patients has been reported to vary between 0.04 and 0.35 m/s. As the gait cycle includes double support phases, where both feet are touching the ground, the speed of one leg during its swing phase may be greater than the mean walking pace. To take this fact into account, the cane forward speed has been designed to be equal to 1 m/s, which is approximately twice the pace reported in Peel et al. (2012). In Murray et al. (1969), the authors collected data from 53 disabled people, and analyzed the load they were applying along the axis of their canes. They reported a mean vertical force of 147 N. In Chen et al. (2001), a decomposition of the load applied to canes by post-stroke hemiplegic patients has been achieved. The results show that the cane bears ~13% of the body weight in the vertical direction and <1% in the posterior-anterior and lateral directions. For a weight of 70 kg, this corresponds to 91 and 1 N, respectively. This gives an idea about the cane design needs in terms of forward velocity and payload.

### 2.2. Mechanical Architecture

**Figure 2:**
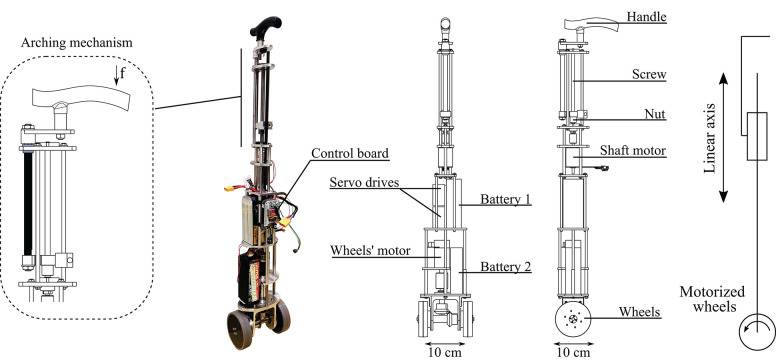
Mechanical structure of the robotic cane. With the arching mechanism (left), a force *f* on the handle results in a torque, inducing friction between the black and the blue pieces. The black piece is arched and the handle can not move downwards.

The cane is shown in Figure 2. It is composed of a telescopic shaft and two wheels; all of them are actuated. Its base consists in a 10 cm square, and the shape becomes thinner while approaching the handle. Its height is adjustable and can vary between 0.85 and 1 m. The shaft translation is ensured by a 100 W EC-i40 Maxon driving a 2.5 mm ball screw mechanism. This ensures nominal velocity and force of 0.16 m/s and 82 N, respectively. The wheels, of 10 cm diameter each, are located at the tip of the cane and are driven together. They are actuated by a 50 W Maxon EC-45 flat motor associated with GP42C gear-head. This gives rise to a nominal velocity and a nominal torque of 1.15 m/s and 40 Nm, respectively. Note that the velocity and force at the cane tip are dependent on the normal force applied to the cane and the friction between the wheels and the ground.

The whole cane prototype weights 5.7 kg. Thus, it is lighter than common robotic walking aid devices or even other stick-like robotic canes, such as that in Phi and Fujimoto (2019). Besides, the batteries, which are the heaviest components, are placed in the lower part. This way, the center of mass of the structure is near the floor allowing to operate the cane from the handle in a light and comfortable way. Note as well that the cane is not lifted from the ground during its operation.

The telescopic shaft is designed based on a reversible ball-screw mechanism. As explained above, the force supported by the latter is 82 N, and thus not sufficient to bear the maximal vertical load applied by a user when leaning on the cane, which approximately equals to 91 N (see previous subsection). Thus, the arching mechanism (see Figure 2 left) is crucial to ensure gait safety. If the user applies a force on the handle, the resulting moment yields friction and prevents any downward motion of the shaft. The arching mechanism makes the upper part of the cane irreversible, without adding any additional weight or bulkiness.

### 2.3. Control Electronics and Sensors

The control architecture is implemented as follows: the cane control is carried out by a BeagleBone board with a sampling frequency of 50 Hz. It communicates via WIFI with two IMUs from X-IO Technologies. One is attached to the cane and provides its angle. The other is attached to the participant impaired leg, providing both its angle and angular velocity. The IMUs provide angles in an Euler representation. Using serial communication, the board acquires the positions of the shaft and the wheels. It then computes and sends the reference velocities to the servo drives. The latter are Solo-Whistle from Elmo. The whole system is powered thanks to two LIPO batteries of a 18.5 V, 7 Ah and a 22.2 V, 4.2 Ah, respectively. They allow a operating time from 1 to 2 days, depending on the frequency of the cane usage. However, the batteries of the IMUs can not withstand more than half a day. This issue is simple to fix. The IMU attached to the cane can be powered by the cane batteries. The one worn by participants can be powered by an external USB charger.

## 3. Synchronization Strategy

Our synchronization strategy is based on the slaving of the cane angle on the impaired leg during its swing, and on the cane immobilization during the stance phase. First, a simple method to detect online the gait phases is provided. Then, experimental evidence is given about the soundness of the method and about the coupling between of the impaired thigh and a conventional cane angle.

### 3.1. Gait Phase Detection Algorithm

When the detection of the gait phase is needed, motion capture and force platforms are appealing solutions if the gait takes place in clinical laboratories. When outdoor motion capture is required, affordable sensors, like accelerometers and gyroscopes, are often used (Mayagoitia et al., 2002). For example, a 3-axis accelerometer held against the sacrum has been used in Evans et al. (1991) to detect heel strikes. In Willemsen et al. (1990), 3-axis accelerometers have been attached to the shank of hemiplegic individuals' impaired legs in order to detect swing phases. The authors of Dai et al. (1996) use tilt sensors in the lower leg to detect the swing phase and deliver electric stimulation. Moreover, in Maqbool et al. (2017) the authors present an approach to real-time detection of mid-swing phase, toe off and initial contact using peaks in the shank angular speed with a wireless gyroscope. In Hwang et al. (2018), the authors propose a method for real-time gait analysis based on a head-worn IMU. The user head vertical acceleration is processed to peak detection since the impact of the foot on the ground at heel strike, and the upward motion during toe off, are transmitted to the head along the body axis. In Martinez-Hernandez et al. (2018), swing and stance phases are detected with a method based on simultaneous Bayesian recognition. The authors use three IMUs attached to the thigh, the shank and the foot, respectively.

**Figure 3:**
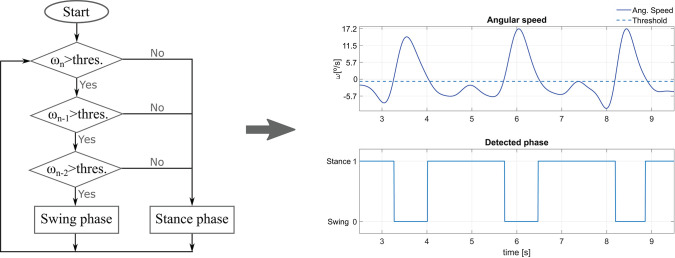
**(Left)** Flowchart of the gait phase detection method. ω denotes the thigh angular velocity, while *n* stands for the sample time. **(Right)** Impaired leg phase detection using the method for an average experiment volunteer.

In our case, the distinction between the stance and swing phase is accomplished with the gyroscope included in the wireless IMU placed on the assisted leg thigh. The angular velocity sign allows to detect whether the leg is in a stance or in a swing phase. The thigh performs an anti-clockwise rotation during the swing phase, and a clockwise rotation during the stance phase. Hence, a threshold on the thigh angle angular velocity in the sagittal plane allows a distinction between them. The detection scheme is showed in Figure 3 left. Once the angular speed has exceeded the threshold during three consecutive samples, a swing phase is detected. Otherwise, the phase is considered to be stance. The number of samples exceeding the threshold to identify the change of phase has been experimentally determined and the measure is aimed to avoid false positives due to noise peaks.

### 3.2. Experimental Validation

The experiments involved six healthy individuals, three males and three females, aged in average 27.7 years old. The study was carried out in conformity with the Declaration of Helsinki of the World Medical Association, and all the participants gave their informed consent. Their gait was altered with a hands free crutch aimed to immobilize injured legs. The impaired leg was simulated by equipping the crutch. They were asked to use a conventional cane in a contralateral way. The participants were equipped with a set of optical markers, so that the motion of their limbs was captured by an OptiTrack system. The cane position and orientation were also captured through the use of extra markers. The experiments began after a 5 min familiarization period. The experiments were composed of series of 3.5-m forward assisted-gait, corresponding to slow, normal, and fast walking paces. The volunteers undergone three tests at each speed.

The mean values (±standard deviations) of these speeds across all the trials and the subjects were respectively 0.23 m/s (±0.06), 0.36 m/s (±0.08), and 0.52 m/s (±0.12).

#### 3.2.1. Validation of the Coupling Between the Impaired Leg Thigh and the Cane Orientation

For each trial, Pearson correlation between the angles of the simulated impaired leg and the cane was computed. For each walking pace, the average of the correlation coefficients across all the trials and participants was computed. The obtained values were *r* = 0.92 (±0.04), *r* = 0.91 (±0.03), and *r* = 0.9 (±0.07) for respectively the slow, normal and fast paces. This shows that the cane angle is strongly correlated with the thigh orientation of the impaired leg.

#### 3.2.2. Validation of the Gait Detection Phases

For each trial, the heel strike and the toe-off ground-truth instants were extracted thanks to the capture motion markers attached to the participants feet. At the same time, the detection technique showed in Figure 3 was used to determine if the impaired leg was in its stance or swing phase. The impaired leg angular speed was obtained by deriving the angle acquired by the motion capture system. The results show that both the heel strike and toe off are detected with reasonable accuracy with respect to the considered ground-truth instants. Thus, the user balance is not threatened (the worst reported difference was in the order of 0.2 s). The method gives good results but can still be tuned more specifically (e.g., by varying the detection threshold) for each user and walking pace to improve the accuracy. Figure 3 right shows an example of the gait phase detection for an average experiment participant.

## 4. Control Implementation

As previously mentioned, the robotic cane should be controlled in order to maintain a constant height of its handle during the whole gait cycle. Moreover, during the assisted leg swing phase, the cane angle should track this leg thigh orientation. In the sequel, the kinematic model of the device is given. Then, the control laws associated to the height and angle servoing are detailed.

### 4.1. Robotic Cane Kinematic Model

**Figure 4:**
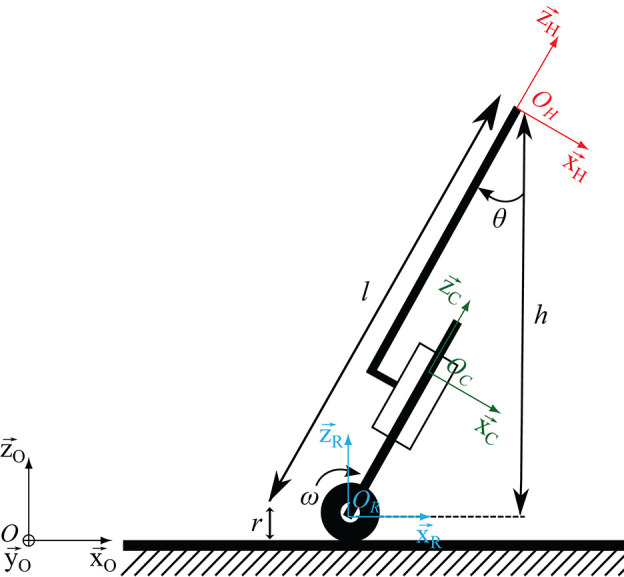
Robotic cane model, ℜ_0_ inertial frame, ℜ_*R*_ the wheel frame, ℜℜ_*C*_ chassis frame, ℜ_*H*_ handle frame.

A sagittal plane kinematic representation of the robotic cane is given in Figure 4. Three coordinate frames are attached to the three bodies composing the cane. ${ℜ}_{\text{R}}=\left\{O_{R},\vec{x_{R}},\vec{y_{R}},\vec{z_{R}}\right\}$ is attached to the wheel. ${ℜ}_{\text{C}}=\left\{O_{C},\vec{x_{C}},\vec{y_{C}},\vec{z_{C}}\right\}$ and ${ℜ}_{\text{H}}=\left\{O_{H},\vec{x_{H}},\vec{y_{H}},\vec{z_{H}}\right\}$ are attached, respectively to the lower and upper parts of the chassis. *O*_*R*_ and *O*_*H*_ represent the centers of, respectively, the cane wheels and handle. *O*_*C*_ is a point belonging to the lower part of the chassis. The length *l* of the chassis is made variable thanks to the motorized axis. The cane orientation in the sagittal plane is defined by the angle θ representing the rotation of ℜ_h_ with respect to the world frame ${ℜ}_{\text{0}}=\left\{O_{0},\vec{x_{0}},\vec{y_{0}},\vec{z_{0}}\right\}$ around $\vec{y_{0}}$. The radius of the wheels and the handle center height are denoted, respectively *r* and *h*.

The cane is assumed to remain in contact with the ground and the wheels to roll without slipping. These two assumptions give rise to mechanical constraints that allow establishing kinematic relationships between the velocity of *O*_*H*_, the cane orientation θ and the wheel rotation speed ω. To establish these relationships, the velocity of *O*_*H*_ is first derived:

(1) $$\begin{array}{l} {\vec{V}}_{O_{H}\in {ℜ}_{\text{H}}/{ℜ}_{0}}={\vec{V}}_{O_{H}\in {ℜ}_{\text{H}}/{ℜ}_{\text{C}}}+{\vec{V}}_{O_{H}\in {ℜ}_{\text{C}}/{ℜ}_{0}} \end{array}$$

Since the upper part of the chassis is translating with respect to the lower part, the following equation holds:

(2) $$\begin{array}{l} {\vec{V}}_{O_{H}\in {ℜ}_{\text{H}}/{ℜ}_{\text{C}}}=\dot{l}\vec{z_{\text{C}}}=\dot{l}\text{ sin }\theta \vec{x_{0}}+\dot{l}\text{ cos }\theta \vec{z_{0}} \end{array}$$

Moreover, it can be written that:

(3) $$\begin{array}{l} {\vec{V}}_{O_{H}\in {ℜ}_{\text{C}}/{ℜ}_{0}}={\vec{V}}_{O_{\text{R}}\in {ℜ}_{\text{C}}/{ℜ}_{0}}+\vec{O_{H}O_{R}}\wedge {\vec{\Omega }}_{{ℜ}_{\text{C}}/{ℜ}_{0}}\\ \;={\vec{V}}_{O_{\text{R}}\in {ℜ}_{\text{C}}/{ℜ}_{0}}-l\vec{z_{\text{H}}}\wedge \dot{\theta }\vec{y_{0}}\\ \;={\vec{V}}_{O_{\text{R}}\in {ℜ}_{\text{C}}/{ℜ}_{0}}+l\dot{\theta }\text{ cos }\theta \vec{x_{0}}-l\dot{\theta }\text{ sin }\theta \vec{z_{0}} \end{array}$$

As the wheels are rolling without slipping, it comes that:

(4) $$\begin{array}{l} {\vec{V}}_{O_{\text{R}}\in {ℜ}_{\text{C}}/{ℜ}_{0}}=r\left(-\omega +\dot{\theta }\right)\vec{x_{0}} \end{array}$$

Putting together equations (1), (2), (3), and (4) gives:

(5) $$\begin{array}{l} \vec{V_{O_{H}\in {ℜ}_{\text{H}}/{ℜ}_{0}}}=\underset{{\dot{x}}_{O_{H}}}{\underset{︸}{\left(\dot{l}\text{ sin }\theta +r\left(-\omega +\dot{\theta }\right)+l\dot{\theta }\text{ cos }\theta \right)}}\vec{x_{0}}+\\ \;\underset{{\dot{z}}_{O_{H}}}{\underset{︸}{\left(\dot{l}\text{ cos }\theta -l\dot{\theta }\text{ sin }\theta \right)}}\vec{z_{0}} \end{array}$$

Equation (5) shows that, as expected, any variation the telescopic shaft length and any rotation of the cane wheels give rise to a displacement of the cane handle.

The control law, that will be given in the sequel, will ensure that $\dot{z}$_*O*_*H*__ and $\dot{x}$_*O*_*H*__ are equal to zero in order to avoid moving the subject hand and threatening balance.

### 4.2. Control Law Structure

To provide the necessary assistance, the cane controller has to fulfill two mains tasks: zeroing the tracking angular error between the cane and the impaired leg while keeping an almost constant height of the handle. The cane kinematics are governed by Equation (5). Thus, this equation constrains the control law.

The controller is composed of two loops: an inner loop aiming at keeping a constant height, and an outer loop dedicated to the tracking of the impaired leg angle. The inner loop should have a shorter response time. This structure allows an easy tuning while respecting the cane kinematics governing law.

The two components of the control law are described below.

#### 4.2.1. Cane Handle Height Control

The cane telescopic axis is controlled to maintain a constant height of the cane handle during the assisted gait. The axis length variation should verify the following equation ($\dot{z}$_*O*_*H*__ = 0):

(6) $$\begin{array}{l} {\dot{z}}_{O_{H}}=\frac{\text{d}}{\text{dt}}\left(l\text{ cos }\theta +r\right)=\dot{l}\text{ cos }\theta -l\dot{\theta }\text{ sin }\theta =0 \end{array}$$

Let us assume that, at the beginning of the gait, the cane handle height is defined by *z*_*O*_*H*0__ in the ℜ_0_ frame.

At the beginning to the experiment, the cane is held vertically. *z*_*O*_*H*0__ is then equal to *l*_0_+*r* in the considered cane orientation. This way, *l*_0_ = *z*_*O*_*H*0__ − *r*, where *l*_0_ is the initial telescopic axis length, at the vertical cane orientation, and *r* the radius of the wheels.

To maintain a constant height regardless of the cane orientation θ, and considering a motionless contact point of the cane, the telescopic axis length must satisfy:

(7) $$\begin{array}{l} l_{d}=\frac{z_{O_{H0}}-r}{\text{ cos }\theta } \end{array}$$

where *l*_*d*_ is a varying set point.

To maintain a constant height, the following control law is implemented:

(8) $$\begin{array}{l} \tilde{\dot{l}}=K_{a}\left(\underset{l_{d}}{\underset{︸}{\frac{z_{O_{H0}}-r}{\text{ cos }\theta }}}-l\right) \end{array}$$

where $\tilde{\dot{l}}$ is the reference velocity sent to the servo drive of the linear axis. *K*_*a*_ is a proportional gain. If $\theta \notin \left\{\frac{-\pi }{2},\frac{\pi }{2}\right\}$, the continuous-time asymptotic convergence of the height adjustment is ensured if *K*_*a*_ is strictly >0. Two *K*_*a*_ values could be assigned depending on the weak leg phase in the gait cycle. The gain is small during the weak leg stance phase in order to limit the linear axis motion and provide a safe support. Moreover, if the force applied by the user is sufficient, the axis may be arched. The gain is higher during the swing phase in order to comply more efficiently with the user hand motion. Hereafter, *K*_*a*_ was chosen equal 3 and 5*s*^−1^ during the stance and swing phase, respectively.

#### 4.2.2. Cane Orientation Control

The control of the cane orientation is achieved through the modulation of the velocity of the wheels. Their rotation influences the velocity of the handle as can be seen in Equation (5). To cancel this influence, the velocity of the wheels should satisfy $\dot{x}$_*O*_*H*__:

(9) $$\begin{array}{l} \omega =\frac{\left(r+l\text{ cos }\theta \right)}{r}\dot{\theta }+\frac{\text{ sin }\theta }{r}\dot{l} \end{array}$$

Since *z*_*O*_*H*__ = *l*cosθ + *r*, and $\dot{z}$_*H*_ = 0 (this is the objective of the telescopic axis control), it comes that:

$$\dot{l}=\frac{l\text{ sin }\theta }{\text{ cos }\theta }\dot{\theta }$$

Equation (9) writes:

(10) $$\begin{array}{l} \omega =\frac{\left(r+l\text{ cos }\theta \right)}{r}\dot{\theta }+\frac{l{\text{ sin}}^{2}\theta }{r\text{ cos }\theta }\dot{\theta }\\ \;=\frac{\left(r\text{ cos }\theta +l\right)}{r\text{ cos }\theta }\dot{\theta } \end{array}$$

The control law of the wheels established to reduce the motion of the cane handle is:

(11) $$\begin{array}{l} \tilde{\omega }=K_{r}\frac{\left(r\text{ cos }\theta +l\right)}{r\text{ cos }\theta }\left(\theta_{d}-\theta \right) \end{array}$$

with $\tilde{\omega }$ representing the speed input sent to the servo-drive driving the cane wheels and θ_*d*_ the assisted limb thigh orientation to be followed. Assuming a correct estimation of the wheels radius, and $\theta \notin \left\{\frac{-\pi }{2},\frac{\pi }{2}\right\}$, the continuous-time asymptotic stability is ensured by choosing *K*_*r*_ strictly positive. During our experiments, *K*_*r*_ was equal to 3.8*s*^−1^.

## 5. Experimental Results

**Figure 5:**
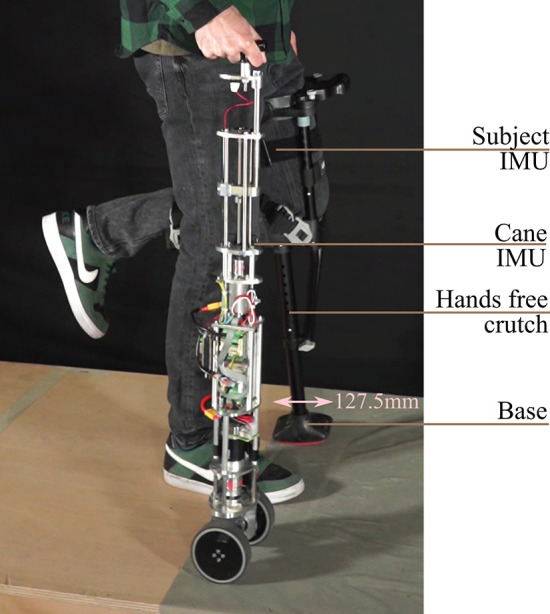
Subject holding the robotic cane. There is a wireless IMU on the user thigh and another on the cane.

In this section, an experimental evaluation of the cane adaptive motion with the gait cycle is carried out. In subsections 5.1 and 5.2, the different tests were performed by a member of the team. In subsection 5.3, the robotic cane was evaluated by a group of volunteers. The experimental context is shown in Figure 5. The participant left leg (that to be assisted) and the cane were equipped with wireless IMUs, so the latter was synchronized with the gait cycle based on the leg motion. The rear and the front of the base of the hands free crutch attached to the left leg were equipped with optical markers. It emulated the foot of the impaired leg. The right hand and the cane tip were as well-equipped with markers. The data recorded by the BeagleBone board were the left thigh orientation and its angular speed, the cane orientation, the velocity of the wheels, the telescopic shaft velocity and the detected gait phase. The cane synchronization with the gait has been assessed using the experimental data obtained thanks to the Optitrack motion capture system in addition to the data logged into cane board memory.

### 5.1. Assessment of the Support Provided by the Robotic Cane

**Figure 6:**
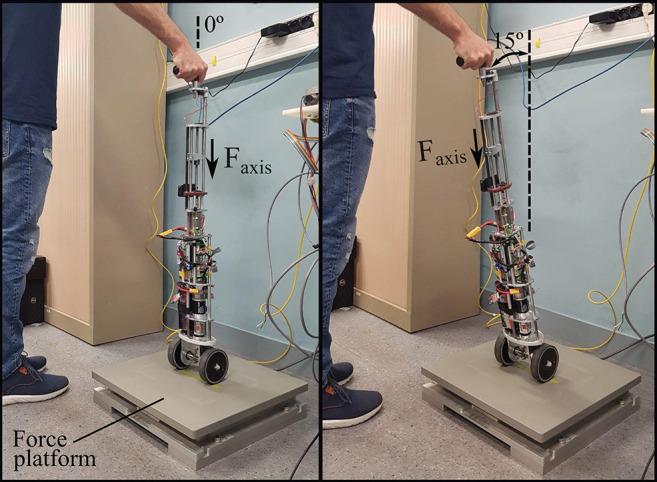
Experimental testing of the forces that the robotic cane can withstand. The cane is placed on a force platform in a vertical position **(left)**, and then inclined by 15° **(right)**. Both motors' velocities were set to zero.

**Figure 7:**
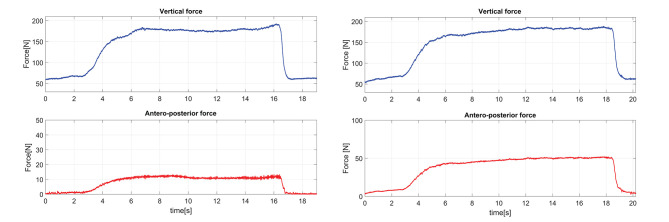
Forces applied on the cane axis with a constant cane length of 0.88 m, an angle of 0° **(left)** and an angle of 15° **(right)**.

In order to assess the ability of the arching mechanism and the wheels' motor to withstand the forces applied on it, the cane was placed on a force platform and the participant was asked to lean on it. The force platform allowed the monitoring of the forces along the axis of the cane frame (see Figure 6). The wheels' motor velocity was set to zero, which is the case when a stance phase of the impaired leg is detected. The shaft motor reference velocity was also set to zero. This setting is the most challenging and corresponds to the phase (d) of Figure 1 bottom. The cane is vertical so the shaft velocity is supposed to be equal to zero while the user is exerting the highest vertical load. The participant applied forces on the cane which was put in two directions corresponding to the vertical 0° and one of 15° (see Figure 7). Note that these forces were artificially high since the purpose of this exercise was to test the cane support performance. One can see that the maximum vertical applied force to the cane was around 180 N, whereas the tangential force varied between 10 and 50 N for the vertical and 15° inclination, respectively. This is in accordance with the design objectives of section 2.1.

### 5.2. Cane Performance Assessment

#### 5.2.1. Metrics for Tracking Performances

Here, the error parameters used to quantify the active cane performance are explained. On the one hand, the cane capacity to track the impaired leg motion is assessed by the Root Mean Square Error (RMSE). It provides insight into the control law tracking performance. On the other hand, the second parameter has been called Mean Distance-to-the-Foot Error (MDFE) and it is a measure of how successful the cane is fulfilling its assistive task, by stopping in the suitable area and providing proper support to the user.

- Angle Root Mean Square Error
  The *Angle RMSE* provides a measurement of the difference between the angles of the active cane and the impaired leg during the assisted walk. It is computed for the swing phases of this leg since it is in these phases when the tracking is active.
  (12) $$\begin{array}{l} Angle\;RMSE={\left[\overset{N}{\underset{i=1}{\sum }}{\left(\theta_{IL_{i}}-\theta_{AC_{i}}\right)}^{2}/N\right]}^{1/2} \end{array}$$
  where θ_*IL*_ and θ_*AC*_ are the impaired leg and active cane angles, respectively, and *N* is the number of samples acquired in the impaired leg swing phases of the test for which the parameter is computed.
- Mean Distance-to-the-Foot Error
  The *Mean Distance-to-the-Foot Error* (MDFE) aims to quantify how good the support given by the cane is when users lean on it. Thus, from the assistance standpoint, this parameter tries to answer to the question: *is the cane well-placed during the impaired leg stance phases?* The MDFE is useful to check if the cane tip is properly located in the walking direction axis with respect to the position of the impaired leg foot.

Note that, as explained at the beginning of section 5, in the experiments the impaired leg is simulated with a hands free crutch (see Figure 5), so that this leg foot corresponds to the crutch distal base. The MDFE is computed as the mean distance from the cane tip to the boundaries of this base. That is to say, for each test sample if the cane tip remains inside the crutch distal base boundaries, the error is zero. On the contrary, if the cane tip stops above or behind the coordinates of the base boundaries, the error is the distance between the cane tip and the base front or the base rear, respectively. Algorithm 1 helps clarify how this parameter is calculated. Let us consider *y* as the axis in the walking direction. *y*_*Ctip*_, *y*_*CBfront*_, and *y*_*CBrear*_ are the arrays with coordinates of the cane tip, the crutch base front, and the crutch base rear. *N* is the number of samples acquired in the impaired leg stance phases.

**Algorithm 1:**
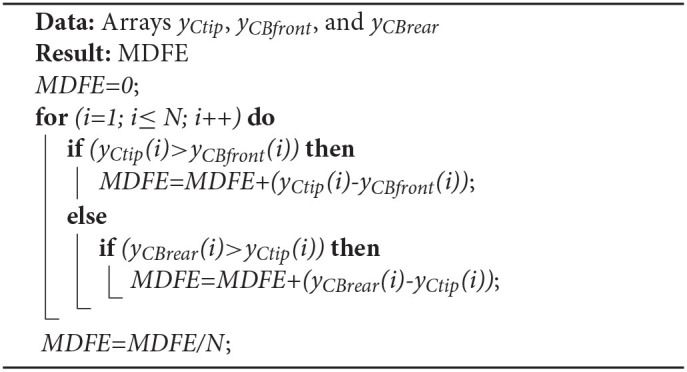
Mean Distance-to-the-Foot Error computation.

#### 5.2.2. Cane Behavior During a Single Step

**Figure 8:**
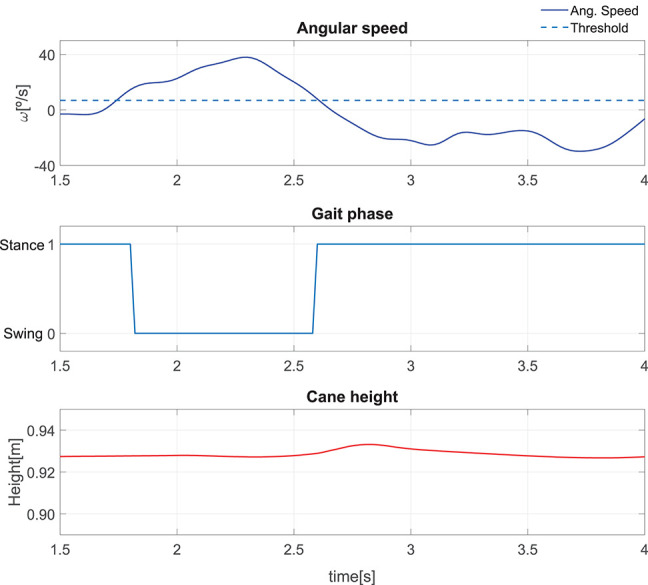
Performance of a single step: angular speed captured by the IMU attached to the impaired leg **(top)**. Gait phase detected using the angle acquired by the motion capture and the method in Figure 3 **(center)**. Cane height **(bottom)**.

**Figure 9:**
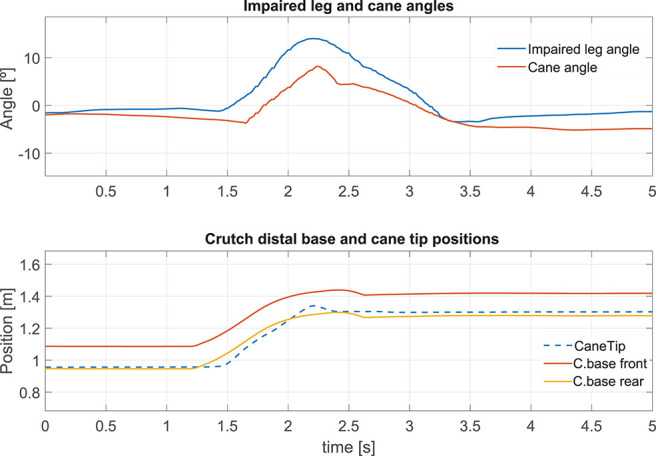
Impaired leg and cane tip angles during a step **(top)**. Position of the crutch base boundaries and the cane tip along the walking direction during a step **(bottom)**.

**Figure 10:**
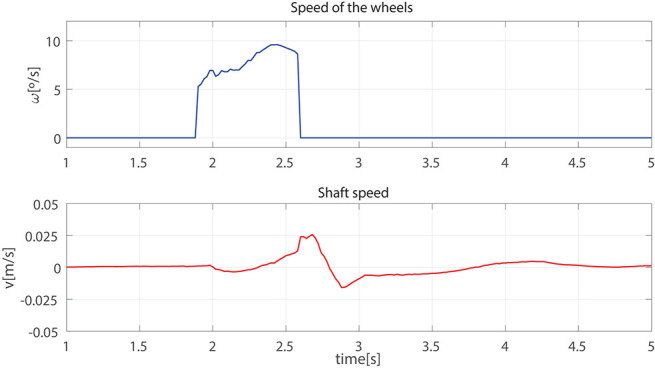
Speed of the wheels **(top)** and the telescopic shaft **(bottom)** observed during a step.

The results presented hereafter show the cane behavior during a step beginning the gait. The subject at rest, performed a 0.25 m step with its impaired leg. Figure 8 compares the phase detection performed thanks to the angular speed provided by gyroscope of the impaired leg IMU with the detection obtained with the motion capture system (ground truth). The comparison indicates good performances of the proposed method. The stance to swing transition (*t* = 1.77 s) is obtained by monitoring the angular speed given by the gyroscope which is multiplied by 100 to make its observation easier. The swing is detected when the threshold indicated in dashed line is crossed as explained in Figure 3. During the swing phase (between *t* = 1.77 s and *t* = 2.58 s), the motion of the wheels is enabled. Figure 9 represents the impaired leg following performed by the cane. The cane tip remains most of the time between the crutch base front and back boundaries. As the angular velocity given by the gyroscope becomes negative, the stance phase is detected as shown in Figure 8. During the whole stance phase, the shaft control maintains the cane handle height practically to its initial value of 0.93 m (Figure 8 bottom) and the motion of the wheels is disabled so as to offer an immobile support point. The evolution of the speeds of the wheels and the shaft is given in Figure 10.

#### 5.2.3. Robotic Cane-Assisted Gait for a Walking Speed of 0.18 m/s

**Figure 11:**
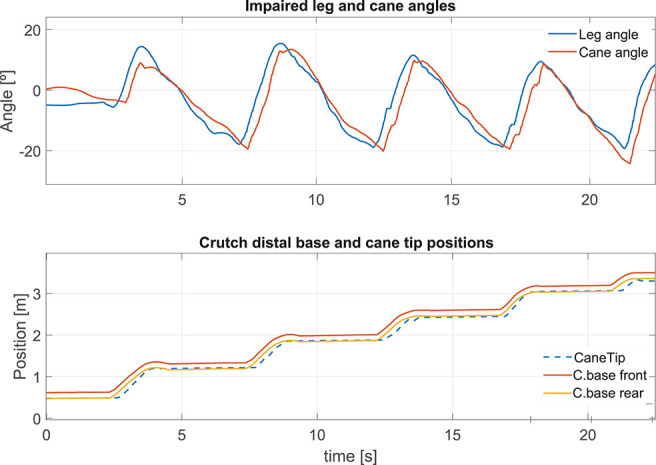
Impaired leg and cane tip angles **(top)**, and positions of the crutch base boundaries and the cane tip along the walking direction **(bottom)** for a speed of 0.18 m/s.

We observed the cane behavior for a slow walking speed of 0.18 m/s. The results of the left thigh following performed by the cane are presented in Figure 11 top. As the wheels' motion occurs during the swing phase, this is the time span in which the error must be computed. The Angle RMSE is 7.81°. Note that the cane angle has a small lag. It is due to the fact that three consecutive samples have to exceed the swing phase detection threshold (see Figure 3) before the tracking starts. The latter accounts for the Angle RMSE increase. Despite the error during the cane following, we noticed that the cane tip remained near the front and back boundaries of the crutch distal base during the stance phases (see Figure 11 bottom). It suggests that, despite starting moving later, the cane quickly shortens the angle difference with the weakest leg and stops at a point where proper support is given. The latter is backed by a little MDFE of 0.0061 m.

**Figure 12:**
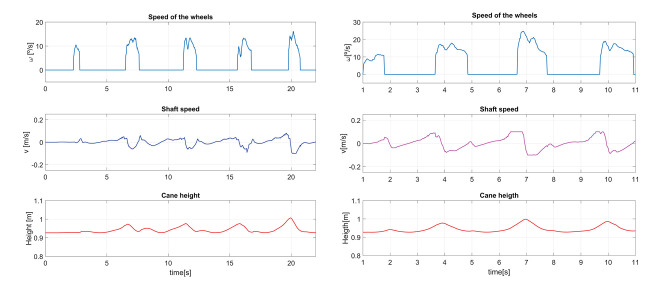
Speed of the wheels and the cane shaft, and cane height for a walking speed of 0.18 m/s **(left)**. The same parameters for a walking speed of 0.35 m/s **(right)**.

The cane handle height was almost maintained at a constant value (Figure 12 left bottom). The wheel and shaft speeds provided in Figure 12 left top and center remained between the speed boundaries implemented in the software. During the stance phase, the cane wheels remained still and the shaft speeds were reduced thanks to a lower gain.

#### 5.2.4. Effect of an Increase of the Walking Pace on the Cane Performances

**Figure 13:**
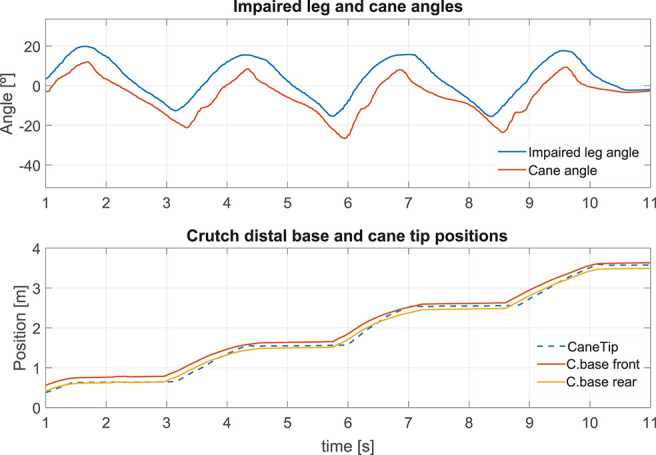
Impaired leg and cane tip angles **(top)**, and positions of the crutch base boundaries and the cane tip along the walking direction **(bottom)** for a speed of 0.35 m/s.

We compared the results presented above with an assisted gait performed with a higher walking speed of 0.35 m/s. During the latter, the impaired leg following performance (Figure 13 top) was slightly lower than that obtained for a walking speed of 0.18 m/s, with an Angle RMSE of 8.57°. Nevertheless, the cane tip position remains inside the crutch distal base boundaries (Figure 13 bottom) with a MDFE of 0.0001 m. Note that this value is lower than that computed for the 0.18 m/s test. It may seem contradictory but it may be due to the fact that the user has felt more comfortable at a higher speed. The accumulated experience in the use of the cane may also affect improving the performance.

In comparison with the walk at 0.18 m/s, a higher request of the shaft was observed in order to maintain the cane handle height near its initial value (Figure 12 right center and bottom). Besides, a light increase of the maximum speed of the wheels was noticed (Figure 12 right top).

#### 5.2.5. How to Manage a Voluntary Hand Motion?

**Figure 14:**
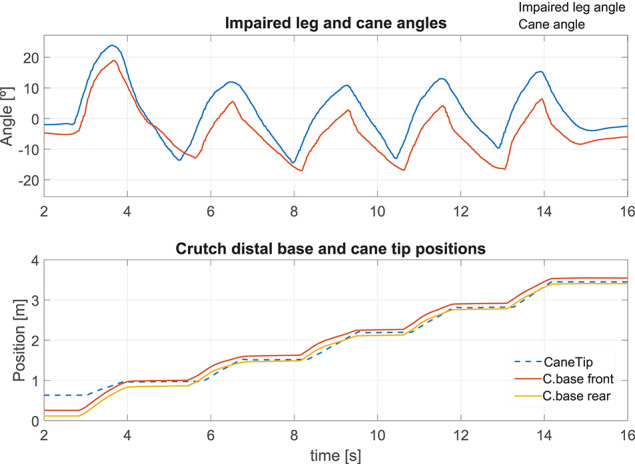
The cane is located ahead of the foot at the start of the walking. Left thigh and cane angles during the test **(top)**. Positions of the crutch base and the cane tip along the walking direction **(bottom)**.

The synchronization strategy assumed the immobility of the hand holding the cane. This is hardly met since the user is not focused on his hand position while walking. Here we illustrate the issue by starting the walk with the cane placed 0.43 m ahead of its user. We show that, if the user brings back his hand toward his body, the synchronization works well. This is depicted in Figure 14. At the bottom, one can see the wrong positioning of the cane at the beginning of the walk, and how the normal functioning has been recovered afterwards.

### 5.3. Assessment of the Cane With Several Participants

The performance of the cane was also assessed through an experiment with six healthy participants, three males and three females (27.5 years old avg.), in conformity with the conditions already specified in the section 3.2 of this article. Although people with real mobility issues are the target population of the active cane, at this stage simulated walking impairment is enough to show the feasibility of the proposal. This way, the participants were equipped with a hands free crutch (see Figure 5) with the purpose of immobilizing their left leg and altering their gait (the crutch distal base is again considered as the impaired leg foot). They used the robotic cane in a contralateral way while equipping optical markers for motion capture. They were allowed to use the robotic cane for 5 min before starting the experiment. The experiment consisted of three tests of 3.5-m forward assisted-gait at their preferred speed. They did not receive any specific instruction on how the cane should be used. Once the tests were carried out, participants were asked informally about their impression after using the cane and none reported comfort issues.

The orientation angle of the robotic cane and the impaired leg were captured. The trajectories of the cane tip and the crutch base were as well-collected. The RMSE was computed for the impaired leg and cane angles during the swing phases, as explained in subsection 5.2.1. In the same way, the MDFE was also calculated. Both parameters, together with the test speed and the number of strides of each test, are listed in Table 1. As can be observed, there is some variability in the values of the Angle RSME, with a minimum of 6.42° and a maximum of 12.31°. The mean, considering all the subjects, is 8.93°, what is not far from that obtained for the tests of subsections 5.2.3 and 5.2.4. The MDFE shows a range that goes from zero (the cane tip remained inside the crutch distal base boundaries in the stance phases) to 0.028 m. The mean MDFE is 0.008 m, what would assure a good support for the user. The speeds are quite different from participant to participant. On the one hand, we find the case of Subject **1**, that presents both the higher speeds and the slowest MDFE. On the other, we have the results of Subject **2**, generally with much lower speeds and the highest MDFE. That supports the idea that, as with a conventional cane, users perform better with the active cane at their preferred pace so a higher speed does not mean necessarily worse walking assistance. This is in line with that observed in subsection 5.2.4.

**Table 1:** Results for the participants involved in the experiments.

**Participant**	**Active cane–Imp. leg** **Angle RMSE (^**°**^)** **(swing phases)**	**Cane tip–Crutch base** **MDFE (m)** **(stance phases)**	**Speed** **(m/s)**	**N^**°**^ of** **strides**
**1**	10.06	0.000	0.21	5
	9.08	0.001	0.22	5
	9.95	0.001	0.21	5
**2**	7.28	0.015	0.13	7
	7.38	0.028	0.13	7
	6.98	0.023	0.17	6
**3**	6.44	0.005	0.13	9
	7.08	0.000	0.14	7
	6.42	0.022	0.12	8
**4**	10.75	0.000	0.19	8
	7.72	0.004	0.16	7
	9.58	0.013	0.16	8
**5**	12.31	0.000	0.21	7
	10.35	0.004	0.18	8
	8.79	0.012	0.16	8
**6**	11.03	0.008	0.12	9
	10.38	0.000	0.12	8
	9.17	0.009	0.10	8
**All Subjects Mean**	**8.93**	**0.008**		

*The parameters in the columns, from left to right, are: subject identifier, Angle Root Mean Square Error (between the cane and the impaired leg), Mean Distance-to-the-Foot Error, test speed, and number of strides per test*.

## 6. Conclusion

In this paper, a control scheme of a robotic cane, which relies on the synchronization of the device motion with the gait cycle, is presented. Its main advantage is its ability to adapt to its user gait parameters. If the step length or the pace change, the cane can automatically adapt its behavior.

The provided control scheme allows robotic canes to provide better assistance than conventional and non-actuated canes. Indeed, the working scheme is planned as follows: during the swing phase of the impaired leg, the robotic cane follows automatically this leg orientation; during the stance phase the cane tip is immobile and provides proper support to the user. Since the robotic cane is not supposed to be lifted during use, all the stumbling risks are eliminated. Besides, the cane handle height keeps as constant as possible to avoid pushing and pulling the user hand during the cane motion. The control strategy feasibility has been shown experimentally.

A short term improvement is to make turning during walking feasible by just rotating the cane, held vertically, around its axis and continuing walking. Detecting a vertical rotation intention will make this improvement possible.

Future work will include the reduction of the robotic cane weight and the improvement of the control law, mainly by reducing the cane angle lag. Moreover, the cane usage time may be extended by using low consumption IMUs instead of the current ones. To improve ergonomics, smartphones can be used to provide the angular information instead of a wearable IMU. Note that smartphones already incorporate an IMU and, normally, people carry theirs with them. One can also think of integrating some new sensors in the cane that enable obstacle detection and other high order functionalities.

Although the proposed concept has been validated through this work, tests with the target population are necessary to confirm its efficacy. They will be planned in the next stage.

## Data Availability Statement

All datasets generated for this study are included in the article/supplementary material.

## Ethics Statement

The studies involving human participants were reviewed and approved by Sorbonne University Ethics Committee. The patients/participants provided their written informed consent to participate in this study.

## Author Contributions

AT-L has been in charge of writing, designing and carrying out the experiments, analyzing the data and results, and drawing the conclusions. RA has contributed with the kinematic model and the original concept. DR has helped with the coding and electronics. WB has collaborated during the whole work providing ideas and supervising. All authors contributed to the article and approved the submitted version.

## Conflict of Interest

The authors declare that the research was conducted in the absence of any commercial or financial relationships that could be construed as a potential conflict of interest.
